# The Effectiveness of an Internet Support Forum for Carers of People With Dementia: A Pre-Post Cohort Study

**DOI:** 10.2196/jmir.3166

**Published:** 2014-02-28

**Authors:** Vicky McKechnie, Chris Barker, Josh Stott

**Affiliations:** ^1^Department of Clinical, Educational and Health PsychologyUniversity College LondonLondonUnited Kingdom

**Keywords:** Alzheimer disease, dementia, caregivers, self-help groups, Internet

## Abstract

**Background:**

The well-being of informal carers of people with dementia is an important public health issue. Caring for an elderly relative with dementia may be burdensome and stressful, and can negatively affect the carer’s social, family, and professional life. The combination of loss, the physical demands of caregiving, prolonged distress, and biological vulnerabilities of older carers may compromise their physical health, increase social isolation, and increase the risk of anxiety and depressive disorders. Caregiver stress is also linked to negative outcomes for the recipient of care and costs to society, including increased nursing home and hospital admissions. Consequently, carer support interventions are an important component of dementia care. Computer-mediated carer support offers a range of potential advantages compared to traditional face-to-face support groups, including accessibility and the possibility of tailoring to meet individual needs, but there has been little research on its effectiveness so far.

**Objective:**

This mixed-methods study examined the impact of a well-respected UK-based online support forum for carers of people with dementia.

**Methods:**

A total of 61 new forum users completed measures of anxiety (7-item Generalized Anxiety Disorder scale, GAD-7), depression (9-item Patient Health Questionnaire, PHQ-9), and quality of relationship with the person with dementia (Scale for the Quality of the Current Relationship in Caregiving, SQCRC), at baseline and again after 12 weeks of forum usage, within a pre-post design. In addition, 8 participants were interviewed about their experiences with using the forum.

**Results:**

There was an improvement in the quality of the relationship with the person with dementia (SQCRC: *P*=.003). There was no change in users’ depression (PHQ-9) or anxiety (GAD-7) over the 12-week study period. Interview participants reported a range of positive experiences and benefits from using the forum. Limited negative experiences were also reported.

**Conclusions:**

Many of the reported experiences and benefits are unique to online peer support. Further research into online peer support for carers of people with dementia is needed to clarify who benefits under what conditions.

## Introduction

There are currently about 800,000 people in the United Kingdom with dementia [1], approximately 1% of the total population. Many are cared for at home by a relative or friend, which can negatively affect the carer’s social, family, and professional life [2]. The rate of anxiety and depressive disorders is increased in carers [3] and caring may also compromise their physical health [3], mortality [4], and ultimately their ability to function as carers.

Therefore, there is a need to find effective ways of supporting carers of people with dementia. The potential of the Internet for this was recognized early on [5] and there now exists a range of multifaceted interventions with elements of networked support [6]. However, conclusions about effectiveness are difficult to draw because of the varied components within each intervention [7]. One common format is the online forum or online support group (we use these terms interchangeably), which provides a number of potential advantages compared with more traditional support mechanisms. These include logistical advantages of carers being able to access support from their homes and cost advantages to service providers.

The limited research specifically looking at online mutual support for carers of people with dementia has generally focused on the content of messages and posts [5,8]. There has been little research examining the outcome of online mutual support for carers of people with dementia, or research that attempts to understand more about how carers find online mutual support to be helpful.

The present study is a mixed-methods evaluation of a well-respected and well-used UK-based online forum for carers of people with dementia. The quantitative component involved baseline and 12-week follow-up measurements of new forum users’ depression, anxiety, and quality of relationship with the care recipient. The qualitative component involved semistructured interviews with new users.

It was hypothesized that after 12 weeks of forum usage: (1) users’ anxiety and depression would decrease and (2) the magnitude of this effect would be correlated with the amount of forum usage. Changes in the quality of the relationship with the person with dementia were also examined. No hypotheses were made in relation to this variable because of 2 conflicting possibilities. Although there is some evidence that the relationship quality might deteriorate over time (eg, [9,10]), it might be anticipated that the forum could increase the quality of the relationship. Therefore, the likely effect is that the quality of the relationship will remain roughly stable.

Qualitative interviews examined participants’ detailed experiences of being on the forum and possible positive and negative outcomes.

## Methods

### Setting

This research was primarily conducted online, with participants accessing the survey through a link on the forum’s home page. There were 8 follow-up face-to-face or telephone interviews. Ethical approval was obtained from the University College London Research Ethics Committee.

### Intervention

**Figure 1:**
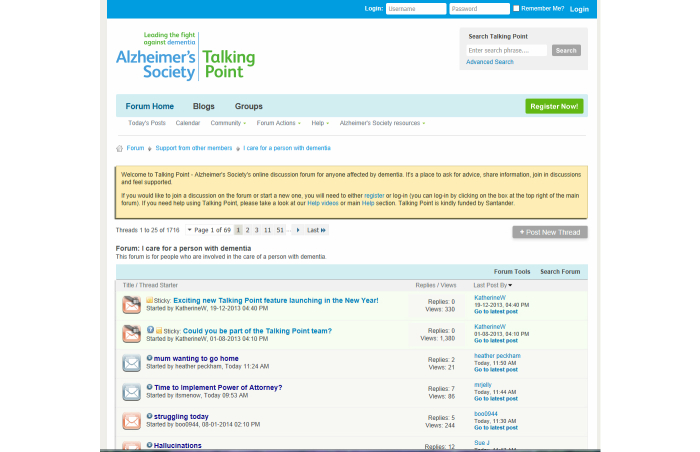
Screenshot of discussion threads on Talking Point’s online forum for carers of people with dementia.

The forum studied was Talking Point, the UK Alzheimer’s Society’s online forum for carers of people with dementia [11]. Its home page describes it as “an online support and discussion forum for anyone affected by dementia. It’s a place to ask for advice, share information, join in discussions, and feel supported.” The forum is well used; on August 20, 2013 there were 873 active members, and at the time of visiting (18:00), there were 444 users online (87 members and 357 guests). It contained a number of different subforums, including “I care for a person with dementia” forum, which was the most active. Figure 1 shows a screenshot of discussion threads on Talking Point.

### Participants

#### Eligibility

Inclusion criteria were that participants had to be (1) a new user on their first visit to the forum, (2) an informal carer for a relative or friend with dementia (ie, unpaid), (3) involved in a significant amount of the care of this person, (4) older than 18 years, and (5) fluent in English.

Additionally, participants were eligible for the qualitative interview if (1) they indicated at baseline that they were interested in being interviewed, (2) they completed the survey at 12 weeks, and (3) they visited the forum at least 6 times over the 12-week study period.

#### Participant Numbers and Response Rate

**Figure 2:**
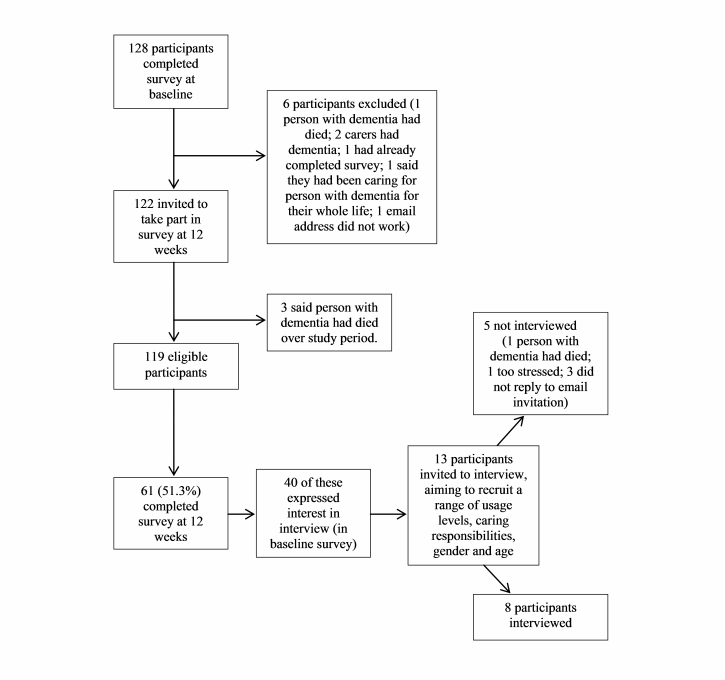
Participant flow chart.

A total of 128 participants completed the first survey between July 25, 2012 and January 9, 2013. In the 6 months between July 1, 2012 and January 31, 2013, 4177 new users registered on Talking Point; therefore, the percentage of potential participants who took part in the research was low (3.06%). Figure 2 shows numbers of participants at each stage in the research process. Table 1 gives demographic information for the 119 baseline participants.

**Table 1:** Participant information (N=119).

Demographic characteristic		Mean or frequency
Age in years (range 22-86), mean (SD)		56 (11.29)
Number of months caring (range 0-408), mean (SD)		44 (56.94)
**Sex, n (%)**		
	Female	99 (83.2)
	Male	18 (15.1)
	Missing	2 (1.7)
**Ethnicity, n (%)**		
	White British	112 (94.1)
	White other	4 (3.4)
	Other	3 (2.4)
**Employment status, n (%)**		
	Employed	58 (48.7)
	Unemployed	19 (16.0)
	Retired	42 (35.3)
**Educational level, n (%)**		
	Primary school	7 (5.9)
	GCSEs/equivalent	22 (18.5)
	A levels/equivalent	16 (13.4)
	University degree	39 (32.8)
	Higher degree/equivalent	18 (15.1)
	Other	9 (7.6)
**Person being cared for, n (%)** ^a^		
	Father	22 (18.5)
	Mother	45 (37.8)
	Partner	38 (31.9)
	Grandparent	3 (2.5)
	Aunt or uncle	3 (2.5)
	Sibling	5 (4.2)
	Mother- or father-in-law	6 (5.0)
	Other	3 (2.5)
**Formal support received, n (%)**		
	General practitioner	24 (20.2)
	Mental health worker or counselor	6 (5.0)
	Another health or social care professional	27 (22.7)
	Memory clinic	22 (18.5)
	Other/not specified	8 (6.7)
	No formal support	55 (46.2)
**Informal support received, n (%)**		
	Friends and/or family	49 (41.2)
	Religious organizations	2 (1.7)
	Recreational groups	1 (0.8)
	Charities, helplines, or forums	12 (10.1)
	Other/not specified	5 (4.2)
	No informal support	51 (42.8)

^a^Figures total more than 100% because 6 participants reported that they were caring for more than 1 person.

### Power Calculation

A recent systematic review [6] found that networked technology interventions for carers of people with dementia had “moderate effects on improving carer stress and depression.” Assuming a medium-small effect size (0.35), a sample size of 67 was required for this analysis [12]. The actual sample size achieved was 61, which gave a power of 0.80 at an alpha of .05 to detect an actual effect size of Cohen’s *d*=0.36.

### Procedure

New users of the forum were invited to take part in the research through an advertisement on the home page, from which they could click on a link to the consent form and survey. An email was also sent to users to alert them to the research, which was also promoted on the Alzheimer’s Society’s Facebook and Twitter pages. At baseline, participants completed 3 standardized measures: the 9-item Patient Health Questionnaire (PHQ-9), the 7-item Generalized Anxiety Disorder scale (GAD-7), and the Scale for the Quality of the Current Relationship in Caregiving (SQCRC). They also answered demographic questions and questions about their role as a carer.

At 12 weeks after completing the first survey, participants were emailed a link to complete the 3 standardized measures again, as well as questions about their use of the forum over the 12-week period. Those who did not complete the second survey within 2 weeks were sent an email reminder.

At baseline, participants indicated if they were interested in an optional interview about their experiences of using the forum. Interview participants were selected according to inclusion criteria and in order to sample a range of different users, in terms of their sex, age, person they were caring for, and length of time they had been caring.

In accordance with the study risk protocol, participants scoring in the severe range on the PHQ-9 or the GAD-7 were advised via email to contact their general practitioner. A total of 49 participants were sent this email.

### Measures

The Patient Health Questionnaire (PHQ-9 [13] is a widely used 9-item measure of depression. Scores of 20 or more suggest severe depression. It has high sensitivity and specificity for diagnosing depression [13], good internal consistency, convergent and discriminant validity, robustness of factor structure, and responsiveness to change [14].

The Generalized Anxiety Disorder scale (GAD-7 [15] is a widely used 7-item measure of anxiety. Scores of 15 or greater indicate severe anxiety [15]. It has good sensitivity and specificity for GAD [15] and is a valid and reliable measure for detecting GAD in the general population [16], as well as social anxiety, panic disorder, and posttraumatic stress disorder [17].

The Scale for the Quality of the Current Relationship in Caregiving 14-item version of the SQCRC (SQCRC-14) [18] asks carers about their relationship with the person that they are caring for, giving equal weight to positive and negative aspects. A higher score implies the presence of warmth and affection and the absence of conflict and criticism in the relationship. The measure has high internal consistency and good face validity [19], but has had little further psychometric investigation.

### Qualitative Interview

The qualitative interview asked participants about their experiences using the forum. It covered what they found more and less useful, how they liked to make use of the forum (eg, whether they preferred to write posts or read other people’s), and whether they felt that it had made a difference to them and their role as a carer. The rationale was to capture the variety of users’ experiences related to both our main outcome variables and also user-defined outcomes. The interview schedule was developed with reference to the literature on peer support, as well as through discussion with the forum’s manager and the charity’s head of evaluation. The manager and volunteer moderators provided feedback during the development of the interview schedule. Seven interviews were conducted over the telephone and one was face-to-face. The interviews lasted approximately 40 minutes.

### Researcher Perspective

It is recommended that the researchers’ perspective be disclosed to enhance the credibility of qualitative research [20,21]. The first author, who conducted the interviews and led the analysis, was a white, middle-class, female clinical psychology doctoral student in her twenties. She had no direct experience of caring for a friend or family member with dementia, but had seen the impact of the caring role on other people and was aware of the challenges and stress that this role can bring. The other 2 authors were white, male clinical psychologists, who were generally favorably disposed toward mutual and peer support. All authors attempted to bracket their preconceptions during the analysis.

### Analysis

Paired sample *t* tests were used to analyze changes in anxiety from baseline to 12 weeks, and depression and quality of relationship for all participants who completed the survey at 12 weeks. Nonparametric correlations were conducted to examine the relationship between forum usage level and changes in outcome.

Semistructured interviews were transcribed verbatim, with all identifiable data removed to preserve anonymity. They were analyzed using thematic analysis [22], taking an inductive, data-driven approach. Interview transcripts were repeatedly reviewed to become familiar with the data and to ensure that information was represented accurately. During this process, an initial list of ideas was generated. These were grouped into codes and then brought together into meaningful themes, which were then checked against initial codes and the overall dataset, and amended in some instances. Finally, themes were organized into overarching domains. Credibility checks [21,23] involved a third party examining sections of analyzed interview transcripts and providing feedback on codes, themes, and domains.

Respondent validation [20] was used as a further credibility check. Each interviewed participant was emailed a summary of the themes generated from their interview and asked to complete a feedback form. Seven of the 8 interview participants responded to this, either by completing the form or simply by replying to the email, and said that they felt that the list of themes was a good summary of their interview.

The quantitative and qualitative data were analyzed separately, using a concurrent analysis approach [24]. This allows each analysis to stand on its own as an independent perspective on the data, rather than have one influenced by the other.

## Results

### Quantitative Analysis

#### Data Preparation

There were no missing data for the 3 main outcome measures at baseline, nor for any participants who completed the survey at 12 weeks. Three participants’ usage data was internally inconsistent. For example, they might have reported that they had visited the forum 10 times, but that they had spent 0 minutes on the forum. Where this occurred, all usage data for that participant was coded as missing. Two additional participants gave unclear answers regarding the amount of time spent on the forum, and this was also coded as missing. The distribution of PHQ-9 scores was positively skewed; therefore, a square root transformation was carried out to conduct statistical tests.

#### Characteristics of Noncompleters and Nonusers

There were no differences in baseline PHQ-9, GAD-7, and SQCRC scores between those who completed the survey at 12 weeks and those who did not (Table 2).

Of the 58 participants whose forum usage data was available, 17 (29%) reported that they had not visited the forum at all over the 12-week study period. There were no differences between baseline scores of users and nonusers on any of the 3 measures (Table 2).

Overall, usage was low (Table 3); 44 (76%) participants visited the forum fewer than 12 times over the 12-week study period. Of those participants who visited the forum, 18 (44%) never replied to any posts, and 20 (49%) never started their own new thread or post.

**Table 2:** Baseline scores on the Patient Health Questionnaire (PHQ-9), Generalized Anxiety Disorder scale (GAD-7), and the Scale for the Quality of the Current Relationship in Caregiving (SQCRC) for those who completed the survey at 12 weeks (completers) and those who did not (noncompleters) and those who used Talking Point (users) and those who did not (nonusers), and pre-post outcome scores.

Baseline score comparison		Group, mean (SD)		*t* (df)	*P*	Cohen’s *d*
		Completers	Noncompleters			
**Completed survey at 12 weeks**						
	PHQ-9	9.75 (6.65)	9.78 (7.35)	0.39 (117)	.69	0.07
	GAD-7	10.38 (6.44)	10.38 (6.65)	0.00 (117)	.99	0.00
	SQCRC	48.61 (9.20)	46.69 (10.08)	–1.09 (117)	.28	–0.12
**Used Talking Point**		Users	Nonusers			
	PHQ-9	9.98 (6.51)	8.88 (6.78)	0.52 (56)	.61	0.15
	GAD-7	9.83 (6.14)	10.71 (6.15)	–0.47 (56)	.64	0.14
	SQCRC	49.00 (9.04)	46.88 (9.86)	0.79 (56)	.43	0.23
**Pre-post outcome**		Baseline	At 12 weeks			
	PHQ-9	9.75 (6.65)	9.23 (6.82)	0.75 (60)	.46	0.19
	GAD-7	10.38 (6.44)	9.72 (6.63)	0.95 (60)	.35	0.24
	SQCRC	48.61 (9.20)	51.36 (9.87)	–3.04 (60)	.003	–0.78

^a^Note that *t* tests were performed on the square root transformed PHQ-9 scores.

**Table 3:** Reported usage levels on Talking Point over the 12-week study period.

Usage variable	Range	Mean	SD
Number of forum visits	0-200	17.43	37.14
Number of minutes spent on forum	0-3000	373.57	589.86
Number of messages/posts written as a reply to someone else	0-400	14.53	54.76
Number of new threads/posts started	0-50	2.67	8.04

#### Pre and Post Outcome Comparisons

Of those who completed the survey at 12 weeks, 16 (26%) had a poor relationship with the person with dementia at baseline (scores ≤42 on the SQCRC), 19 (31%) fell in the severe range for anxiety on the GAD-7, and 6 (10%) fell in the severe range for depression on the PHQ-9.

There was a difference between SQCRC scores at baseline and scores at 12 weeks, but not on the PHQ-9 or GAD-7 (Table 2). There is insufficient psychometric data to evaluate the clinical significance of the improvement in SQCGR scores, but according to the scale developers [19], both the pre and post means are in the good range of greater than 42.

To investigate the hypothesis that users’ outcomes are related to their level of forum usage, nonparametric correlations between outcome and usage were examined for the subsample of 40 participants who said that they had visited the forum at least once over the 12-week study period. Only 1 of these 12 correlations was statistically significant; specifically, between total time on the forum and reduction in PHQ scores (Spearman rho=.356, *P*=.03).

#### Individual Change

Table 4 shows reliable change analysis at the level of individual participants [25] (reliabilities for the 3 measures were obtained from PHQ-9 [26]; GAD-7 [16]; SQCRC [19]). Most participants showed no reliable change.

**Table 4:** Reliable change analysis for Patient Health Questionnaire (PHQ-9), Generalized Anxiety Disorder scale (GAD-7), and the Scale for the Quality of the Current Relationship in Caregiving (SQCRC).

Measure	Change, n (%)		
	Reliably improved	No change	Reliably deteriorated
PHQ-9	9 (15%)	48 (79%)	4 (7%)
GAD-7	11 (18%)	41 (67%)	9 (15%)
SQCRC	5 (8%)	55 (90%)	1 (2%)

### Qualitative Analysis

From the 61 participants who completed the 12-week survey, 40 expressed an interest in being interviewed and 13 were invited (with the aim of having a variety of backgrounds and forum usage). Of these, 8 were able to be interviewed. Table 5 gives their characteristics.

Thematic analysis of the 8 semistructured interviews resulted in 18 themes across 3 domains (Table 6). Each domain is discussed subsequently, and illustrative quotes can be found in Table 6.

**Table 5:** Interviewee information (n=8).

Participant number	Demographic information	Caring situation
1	Female; age 51 years; white British	Caring for husband (age 53 years) who has frontotemporal dementia. Participant and her husband live together. Caring for approximately 21 months at time of interview.
2	Female; age 48 years; white British	Caring for mother who has a mixed diagnosis of vascular dementia and Alzheimer disease. Mother does not live with participant. Caring for approximately 29 months at time of interview.
3	Female; age 43 years; white British	Caring for father who has dementia and lives locally. Caring for approximately 28 months at time of interview.
4	Male; age 70 years; white British	Caring for wife who has vascular dementia. Participant and his wife live together. Caring for approximately 33 months at time of interview.
5	Male; age 84 years; white British	Caring for wife who has Alzheimer disease. Caring for approximately 30 months at time of interview. Wife moved into care home 1 year previous.
6	Female; age 69 years; white British	Does not consider herself a carer because husband is in the early stages of Alzheimer disease and does not require significant levels of support. At time of interview, it was approximately 29 months since memory problems began.
7	Female; age 63 years; white British	Caring for mother-in-law who lives locally and has dementia. Caring for approximately 12 months at time of interview.
8	Female; age 61 years; white British	Caring for husband who has dementia. Participant and her husband live together. Caring for approximately 18 months at the time of interview.

**Table 6:** Summary and illustration of domains and themes from the thematic analysis.

Domain and theme		Prevalence^a^	Illustrative quotations
**Social similarity**			
	I am not the only one going through this	Typical	“Every time I realize ‘that’s me, that’s me! I’m going through that, that’s me!’ I can relate to so much of what’s on there.” [P1]
	Reduced isolation and loneliness	General	“All of a sudden, I’m in the flat on my own. And I have nobody to talk to...so the only thing I’ve got now, really, is Talking Point” [P5] “Before my son introduced me to that [Talking Point] I felt that I was on my own, even though, like I said, I have fantastic support from the help service and family and everything, it still, I still felt alone.” [P1]
	Normalizing	Typical	“I’m feeling like I want my dad to die, because I don’t want to see him go through this, and he’d be happier. And then you feel full of guilt, but you go on [to Talking Point], and you’re not the only one feeling that, or you’re not the only one that has felt that. And it makes you feel OK, normal.” [P3]
	Other users have experience and are therefore understanding	Typical	“And that is the great strength of it. Everybody on Talking Point has hands-on experience of dealing with dementia. And they make allowances for you, as they did for me.” [P5]
	Being able to share and let off steam	Typical	“If nothing else, it’s purely somewhere to let off steam.” [P4]
	Other users are worse off than I am	Typical	“My goodness, some people are dealing with some incredibly difficult situations...the situation we’ve got at the moment is not that difficult, we ought to be able to work around it.” [P7] “If I’m online and I’m reading other people’s posts and it’s quite distressing, you know, it’s quite easy to get distressed by it. So there are times when I just don’t go on it because I think, oh, it’s too painful, really, for me.” [P2]
	Not necessarily needing to post to benefit	General	“I think it’s more useful for me to read other people’s experiences.” [P6]
	Being able to give advice and support to other users	General	“It’s nice to be giving something back for the information that you’re getting. Or being able to support people as the support you receive.” [P3]. “It’s helpful in that you think, well, at least you know slightly more than someone else...It gives you a little bit of a boost to know that you can help somebody.” [P6]
**Unique aspects**			
	I can ask questions and get the support that I might not be able to get, or might not want to get, elsewhere	Typical	“It’s not the kind of thing you would read in a leaflet that you pick up anywhere.” [P6] “Me personally, I don’t want to go sit in front of a—what would you go sit in front of?—a doctor, or a whoever. I don’t want to talk that way.” [P3]
	I can get information and advice that I would not know where else to get	Rare	“For someone who is totally ignorant of Alzheimer’s it has answered questions that I don’t know where I would have got the answer from.” [P6]
	Control	Typical	“I can go for months without bothering with it...I’ll utilize Talking Point when I need to. That maybe sounds a bit selfish, but I think that’s the advantage of it for me.” [P2] “You’re not seeing what it’s going to be like, you’re not seeing, you’re not hearing what it’s, you read what you want to read.” [P3] “I find it’s very easy to navigate to and from the titles to pick up things that might be useful.” [P7]
	Anonymity	Typical	“What I say there, I am opening up my heart to a very large extent and I am opening myself up and being totally honest about my feelings, and in some cases I wouldn’t want other members of my family, for example, to see some of these things...I wouldn’t want them to know exactly how I feel about certain things. Because when you are with other people you never really uncover your true self, do you?” [P5] “I suppose it’s when you’re sat at home thinking, ‘oh crikey’” you know, someone thinks that’s really awful. And it puts you in a bit of a panic, but I do think the moderators help because they come back with...solutions.” [P2]
	Immediate access and responses	Variant	“Now if I have something that is worrying me, I know that I have immediately got someone to talk it over with.” [P8]
	No time restrictions	Variant	“I think professional support is generally very time limited. And Talking Point isn’t time limited.” [P2]
	Geography is unimportant	Variant	“Particularly with me being mobile around the world as well as the country, it’s offered a support that I wouldn’t have had otherwise.” [P7]
**New learning**			
	Practical learning and information	General	“Off Talking Point, someone said that animals are really good. So I got him a kitten...It was absolutely brilliant, and I thought ‘thank God for Talking Point’ because when my mum had to go out, he had the kitten to talk to, and the kitten to play with, and the cat even now.” [P3]
	Learning how the dementia might progress and what to expect	Typical	“That happened 3 times in the last few weeks, where [husband] hadn’t known where he was. And it has been stressful for him. But had I not known about it, I might have worried more. But having read about it [on Talking Point], I thought, mmm, yes, things do happen.” [P6] “When you’re reading it, you’re thinking, ‘I’ve got all this to come,’ but at the same time, you’re planning.” [P3] “I now look for the kind of problems that other people are having, that I am not having...And so that saddens me in a way that I possibly know more and am looking for things, and really I shouldn’t be.” [P6]
	Developing a better understanding of the person with dementia, and consequently becoming a better carer	General	“I don’t reason with her any more, I agree, and I can steer the conversation, and I know the kinds of things to say and the kinds of things to stay away from. And I think I’m a much better person for Talking Point. A much better companion for my wife, I know that.” [P5]

^a^General: theme applies to all or all but one of the participants (7-8); typical: theme applies to more than half of the participants (5-6); variant: theme applies to up to half of participants (3-4); rare: theme applies to 1 or 2 participants (1-2).

#### Domain 1: Social Similarity

All interview participants emphasized the importance of the forum being for people who are in the same situation—caring for someone with dementia. For the most part, this was considered to be a great benefit, although some also noted disadvantages. Six participants spoke about connecting with other carers on the forum, and their comfort in knowing that “I am not the only one going through this.” Seven participants noted that being able to use the forum reduced their isolation and loneliness. Some had experienced isolation as a result of their caring role, whereas most participants who felt isolated and alone had social support but still felt alone before joining the forum. Six participants noted the normalizing effect of the forum. Six participants reflected that because other users had experience with dementia they were understanding about the struggles that forum users face. This also meant that background information did not need to be explained to others on the forum. Five participants reflected on the value of the forum in being able to “share and let off steam” with others. Several participants commented that other users of the forum were in a worse situation than they were, which made 3 participants feel more positive about their own situation. However, for 3 interviewees, reading other users’ posts had been distressing at times. Seven participants noted that because everyone on the forum was a carer of someone with dementia, they did not necessarily need to post to benefit, and that simply reading the posts was useful. Seven said that they had been able to give advice and support to other users of the forum. For some, this helped people to feel that they were giving back to the forum. For others, it also showed them that they had valuable knowledge to pass on.

#### Domain 2: Unique Aspects

All 8 participants compared the forum to other sources of information and support. Sometimes this was explicitly discussed, but more often it was implied by the advantages the forum offered them. Six participants commented that they could ask questions and get the support that they might not be able to get elsewhere or that they might not want to get elsewhere. Two commented that they would not know where else to get information and advice. Five participants reflected that through using the forum they had control, including control over frequency of usage and, to a certain extent, the ability to avoid posts that were too upsetting. Many also discussed only reading posts that were of interest or personally relevant. Four participants commented on the benefits of anonymity on the forum, including being able to both be more open and honest and to discuss problems that might be uncomfortable to discuss face-to-face. Four participants noted that they had seen inappropriate or judgmental posts on the forum, which may be another consequence of the forum’s anonymity. One participant (P2) had received replies from other users who “put something on about how disgusting it [a post she had made] was.” She and others reflected that the forum’s volunteer moderators were generally helpful in these situations. Three participants reflected that the forum allowed immediate access and response. Three others commented that it could be accessed 24-hours a day for as long as need be. Three participants noted that geography was unimportant. Two participants had lived or currently lived outside the United Kingdom and both reflected on the benefits of the forum for them.

#### Domain 3: New Learning

All participants described learning new information, and many said that what they had learned on the forum had helped them to become better carers. All 8 participants commented that the forum had provided them with practical advice and information. This ranged from information about Attendance Allowance (a government benefit for people with disabilities), to information about how to deal with people with dementia turning on an empty microwave, to one participant buying her father a cat. Five participants noted that they learned about how the dementia might progress and what to expect. For some, this meant that when certain events did occur, they were more prepared for them and less anxious when they did occur. For others, this information meant that they felt forewarned and, therefore, forearmed in terms of the next stages of the dementia. One participant expressed sadness that through what she had read on the forum, she had started to look for certain behaviors in her husband. Seven participants reflected that they developed a better understanding of the person with dementia and consequently became a better carer.

## Discussion

### Principal Findings

There was an improvement in the quality of the relationship with the person with dementia. There was no change in users’ depression or anxiety over the 12-week study period in contrast to other studies of the outcome of online support [27]. Interview participants reported a range of positive experiences and benefits from using the forum. Limited negative experiences were also reported.

The hypotheses that after 12 weeks of forum usage, users’ anxiety and depression would decrease were not supported, although depression and anxiety levels did not increase. This may indicate no effect of forum usage. However, interpretation is dependent upon understanding of the normal expected trajectory of carer mood, and this is complicated because models predict anything from deteriorating to increasing psychological health over time [28]. Additionally, the progression of psychological distress in carers is affected by a range of factors, including intrinsic variables, such as carer gender [29], and more contextual factors, such as the relationship between carer and care recipient [30], which varied across the sample. It may also be that participants’ low usage of the forum accounts for these findings because there is evidence of a dose-response relationship in online forums for mental health conditions [31].

Qualitative results provide some insight into how the forum may have improved the carer’s relationship with the person with dementia; for example, through carers learning more about how to interact with someone with dementia and feeling as though the information that they had gathered helped them to become a better carer. Many participants’ forum usage levels were low, and it is possible that being aware of the forum as a resource that was available should they wish to use it was enough for some participants to improve the quality of their relationship with the person that they were caring for. In addition, it is possible that those who signed up to the forum were individuals who had made the decision to learn and develop as much as possible in their role as a carer, and these individuals were motivated to have a good quality relationship with the person with dementia. It is also possible, however, that the improvement in scores on this measure simply represents regression to the mean.

For all 3 measures, most participants’ scores neither reliably improved nor reliably deteriorated. More participants reliably improved than reliably deteriorated. Although the present study only covered a 12-week period, this finding is encouraging given that relationship quality is likely to deteriorate over time [9,10]. Research into the trajectories of anxiety and depression is more mixed [28], but this is also an encouraging finding in relation to these measures.

The 8 carers interviewed were generally very positive. Their reasons for using the forum echo previous research, which found that the primary functions of such groups are to exchange information, connect to others, and to obtain emotional support [32].

For interviewees, the fact that other forum users were in a similar situation to them had a range of benefits. Carers of people with dementia frequently report feelings of isolation and inadequate social support [33], but interview participants reported that the perceived similarity of other users reduced isolation and loneliness, allowed them to “let off steam,” and feel more normal and understood. These experiences, which correspond to Yalom and Leszcz’s [34] therapeutic factors in group therapy (universality, altruism, guidance, imparting information, and catharsis) are similar to those reported in a study of mental health service users’ information needs [35].

Several interviewees noted that seeing that others were worse off helped them to feel better about their own situation. No interviewee mentioned comparing themselves to individuals better off than them. Benefits of downward comparison have been seen found in online support groups for heath conditions [36,37]. However, for several participants, reading the stories of other users who were in worse situations was distressing.

Interviewees reported deriving benefit from helping others, in-line with the helper therapy principle [38], which is unique to peer support. Providing help can increase feelings of competence, equality, social usefulness, independence, and social value, and allows individuals to view themselves as having strengths as well as needs [39]. The reported benefits of lurking (using the forum without actually posting) are echoed in another study [40] that found lurkers on an online support group reported a range of benefits indicating that reading messages may be as beneficial as interacting with the group.

Several interviewees reflected that the type of information and support available on the forum was either not available to them elsewhere or that they would not wish to obtain it elsewhere. The forum may either work as a complement to other services, or offer a service to people who do not access other services. A number of interviewees were not the main carer of the person with dementia; therefore, perhaps, more traditional services are not available to them.

The forum’s anonymity provided some interviewees with the freedom to say more than they would be able to otherwise. This online disinhibition effect [41] brings the advantage that users may be more open about their feelings allowing access to emotional support. However, several interview participants also considered some posts to be judgmental or inappropriate. Helpful moderators (as found on Talking Point) are important to ameliorate this more toxic online disinhibition effect [41]. Additionally, some participants said that the forum allowed them to read only the posts that they wanted to, allowing them to avoid distressing posts.

Several participants noted that reading posts helped them to learn more about progression of dementia and to be better prepared for future problems. However, others felt that some posts were too distressing to read. With a disease that involves worsening of symptoms over time, more information and less uncertainty about the future can provide relief, but it can also cause distress and more information is not always positive. This tension has also been found in support groups for people with motor neuron disease and their carers [36].

Participants also noted that the forum taught them more about how to interact with someone with dementia and to become a better carer or companion. Consequently, the forum may have benefits for the person with dementia through improved care.

There is an apparent discrepancy between the positive qualitative experiences of the site and the modest pre-post changes. This may be because of the contrast between psychological outcome variables measured quantitatively and the qualitatively reported benefits, which were largely related to general therapeutic group factors [34] as well as to practical advice and learning. This raises questions about what carers hope to gain from the site and provides useful information about outcome measures for future studies, a contentious issue in this field [42,43].

### Limitations

There were several limitations to the study. There was a low usage rate that affected the ability to reliably assess the impact of the forum and skewed the data. However, this may reflect real-life usage and, as a number of participants noted, a benefit was being able to visit the forum as little or as often as desired. Additionally, inclusion criteria for interviewees excluded those who used the forum less or disengaged from using the forum. Their views may have differed from those who were interviewed, but those interviewed still provided useful information about people who do engage with the forum, of whom there are many. Only 8 participants were interviewed, which is a relatively small number, and it is possible that a larger sample of interviewees would have provided different or additional data. Whether these 8 interviewees’ experiences reflect the experiences of the wider group of forum users is difficult to ascertain.

Participants received various levels of additional formal and informal support in their roles as carers, which may further complicate and limit the scope of conclusions that can be made about the specific role of the forum. In addition, only approximately 3% of possible participants took part in the research. These sampling issues inevitably limit the extent of conclusions that can be made, and mean that findings from the quantitative data, in particular, are tentative. The lack of a control group in the present research means that these outcomes cannot be compared to a group of carers who did not have access to Talking Point; therefore, findings cannot necessarily be attributed to forum usage as opposed to other factors. Future studies would benefit from using a randomized controlled design.

Finally, the caregivers in our sample tended to be late in their caring trajectory (adult child vs spouse caregivers) with the median age suggesting likely sandwich generation carers. These caregiver variables have systematic effects on caregiver mental health outcomes [44]; the number of participants scoring in the severe range for depression was relatively low [45] although this was not the case for anxiety [46]. Given these sample characteristics and evidence of low depression rates, caution is needed when generalizing the results to the wider population of caregivers.

### Implications

The qualitative data indicated unique benefits from peer support/group therapy, such as not feeling alone and feeling understood through shared experience. Some of these benefits are unique to online support, such as being continually and flexibly accessible as well as enabling honesty within an anonymous online environment. Therefore, clinicians may wish to direct carers to such online peer support forums. In an economic climate in which services are often being reduced, online peer support is likely to become more prevalent and may be the only support that some carers receive. It is important that this area continues to be researched so that carers can derive maximum benefit from online peer support forums.

Future research could investigate further what outcomes are important to carers and specifically consider these outcomes in the evaluation of online carer support interventions. In addition, more research needs to be conducted into whether different types of carers (eg, according to gender and ethnicity) derive different types or levels of benefit from online peer support forums because previous research (eg, [47,48]) suggests that interventions do not affect carers uniformly. In order that carers receive the best support possible, it would also be worthwhile investigating whether there are certain types of support that work well when offered in conjunction with online peer support and vice versa. Interventions for carers of people with dementia are often most effective when they are multicomponent in nature [49,50], although this makes evaluation and attribution of any observed effects more complex [50].

## Abbreviations

GAD-7: 7-item Generalized Anxiety Disorder scale
PHQ-9: 9-item Patient Health Questionnaire
SQCRC: Scale for the Quality of the Current Relationship in Caregiving
